# HMOS 2’FL and 3FL prevent house dust mite induced proinflammatory cytokine release *in vitro* and decrease specific IgE production in a murine allergic asthma model

**DOI:** 10.3389/fnut.2025.1491430

**Published:** 2025-02-19

**Authors:** Marit Zuurveld, Janna W. M. de Kleer, Alinda J. Berends, Manou M. Kooy, Ingrid Van Ark, Thea Leusink-Muis, Nienke Kettelarij, Gert Folkerts, Johan Garssen, Belinda van’t Land, Linette E. M. Willemsen

**Affiliations:** ^1^Division of Pharmacology, Faculty of Science, Utrecht Institute for Pharmaceutical Science, Utrecht University, Utrecht, Netherlands; ^2^Danone Research and Innovation, Utrecht, Netherlands; ^3^Center for Translational Immunology, University Medical Centre Utrecht, Utrecht, Netherlands

**Keywords:** advanced *in vitro* models, allergic asthma, human milk oligosaccharides, mucosal inflammation, allergy prevention

## Abstract

**Introduction:**

Allergic asthma is characterized by sensitization to airborne allergens like house dust mite (HDM). Human milk oligosaccharides (HMOS) are linked to improved immune maturation and potentially alleviate allergy development.

**Methods:**

A human *in vitro* model for crosstalk between bronchial epithelial cells (BECs), monocyte-derived DCs (moDCs) and T cells, during HDM exposure, was established. The immunomodulatory effects of the HMOS 2′-fucosyllactose (2’FL) and 3-fucosyllactose (3FL) were investigated in this *in vitro* model and subsequently in a house dust mite-induced allergic asthma murine model.

**Results:**

HDM exposure during BEC-DC coculture enhanced type 2 instructing TSLP, while reducing regulatory TGFβ secretion. Coculture of HDM-primed DCs with T cells enhanced IL4 secretion. 2’FL or 3FL preincubation prevented HDM-induced TSLP and IL8 release from BEC-DC. HDM-allergic mice receiving a 1% 2’FL or 0,5% 3FL supplemented diet both had lower serum levels of HDM-specific IgE compared to mice fed control diet. In conclusion, a human *in vitro* coculture model for HDM-induced BEC-DC activation and subsequent type 2 T cell response was established. 2’FL or 3FL preincubation of BEC-DC prevented HDM-induced activation and modified downstream T cell responses *in vitro*. Both HMOS reduced HDM-specific IgE in a murine model for HDM allergic asthma, but did not protect against airway inflammation.

**Conclusion:**

Here, we describe an *in vitro* human airway mucosal HDM sensitization model as relevant tool to reduce use of animals in studies aiming to prevent HDM allergic asthma. Both *in vitro* as well as *in vivo*, HMOS were found to drive away from a type 2 immune signature, paving the way to further investigate the potential allergy preventive effects of fucosylated HMOS.

## 1 Introduction

Allergic asthma is common in Westernized countries, with as many as 1 in 10 children and 1 in 12 adults affected (1). A total of 300 million people worldwide is affected by the disease. Allergic asthma is characterized by sensitization to airborne allergens, which leads to T helper 2 cell (Th2) mediated airway inflammation and asthma symptoms (2). The most common allergens in allergic asthma are derived from house dust mite (HDM), animal dander, cockroaches and fungi (3). Sensitization to allergens mostly occurs in children within the first year of life, often accompanied by atopic eczema (1). These children are generally more susceptible to develop food allergy, as well as allergic rhinitis and allergic asthma later in life (4). There is no curative treatment available for allergic asthma and medication generally consists of a combination of inhaled corticosteroids and short-or long-acting β2-adrenergic agonists. Allergic asthma might lead to chronic inflammation of the respiratory tract in which bronchial epithelial cells (BEC) and innate and adaptive immune cell activation contribute to type 2 inflammation, tissue remodeling and bronchial hyperreactivity (BHR) (1). Symptoms include shortness of breath, wheezing, coughing, and chest tightness (5).

Allergic sensitization has been linked to decreased epithelial barrier function. Disruption of this epithelial barrier, can lead to increased sensitization as allergens can enter the lamina propria more easily, leading to increased exposure to immune cells (6, 7). Thus, airway epithelial integrity is crucial in preventing sensitization and serves as a first line of defense against unwanted intruders. Besides being a physical barrier, the epithelium expresses pattern recognition receptors such as protease-activated receptors, toll-like receptors (TLR), and C-type lectin receptors, which are involved in allergen binding. Furthermore, several allergens found in HDM, including Der p 1, have protease activity (8) disrupting epithelial tight junctions (9, 10). Binding of allergens to epithelial receptors leads to activation of these epithelial cells and the release of alarmins, such as thymic stromal lymphopoietin (TSLP), interleukin (IL)25 and IL33 (11). These alarmins act together with chemokines to activate and/or recruit DCs, innate lymphoid cells type 2 (ILC2) or eosinophils, which promote the inflammatory response (1, 8, 12).

Uptake of allergens by antigen-presenting cells (APCs) occurs through sampling of the airway lumen by dendritic cells (DCs) or through allergens crossing the bronchial epithelium into the underlying lamina propria. Following uptake of the allergen and instruction via epithelial derived mediators, DCs travel to the mediastinal lymph nodes to present the captured allergen within their MHC class II receptor (MHCII) to the T cell receptor (TCR) on naïve Th cells which may leads to differentiation into Th2 cells in the case of allergic sensitization (12). Allergen-specific Th2 cells activate allergen-specific B cells, which leads to class switching of the B cells towards immunoglobulin E (IgE) producing plasma cells. Secreted allergen-specific IgE binds to the high affinity receptor FcεRI present on mast cells. At a second encounter with the allergen, allergen-specific IgE on the surface of mast cells crosslinks by binding to the allergen, leading to mast cell degranulation (13, 14). Degranulation involves the release of pro-inflammatory mediators, which induce vasodilation, bronchoconstriction, and eosinophilic airway inflammation which becomes chronic when being uncontrolled (15).

As treatment of allergic asthma currently mainly consists of symptom suppression, preventing the development of allergic asthma has acquired scientific interest. The World Health Organization recommends exclusive breastfeeding for the first 6 months of life as it has overwhelming benefits for both mother and infant (16), including potential protection from allergic asthma development (17). Human milk oligosaccharides (HMOS) are the third largest solid component of breastmilk after lipids and lactose, present in concentrations of up to 25 g/L in colostrum and 5–15 g/L in mature milk (18–20) and are thought to benefit immune maturation and protect against infections (14, 21). HMOS are resistant to low gastric pH and cannot be digested by humans. However, they are substrates for bacterial fermentation in the large intestine (22, 23). In addition, a small amount of ingested HMOS can be traced back in the blood and urine of the suckling infant (24–26). HMOS directly bind to pathogens, promote growth of beneficial bacteria, can be used to produce bioactive metabolites like short chain fatty acids (SCFA) and act directly on receptors from epithelial cells and immune cells (14). Therefore, differential effects from specific HMOS can be postulated in preventing the development of allergic asthma.

All mammalian milk contains oligosaccharides, but this is generally found in a concentration 10–100 fold lower than in humans (27). Over 150 different HMOS structures have been identified, and are present in human milk (19, 28). Variety in the produced HMOS substantially differs per individual. Factors such as period of lactation or polymorphism of the Lewis and Secretor genes can influence the variety and quantity of HMOS in breastmilk (19). Neutral HMOS account for 75% of the total HMOS in human milk. All women can secrete the neutral fucosylated 3-fucosyllactose (3FL), independent of the expression of the Secretor or Lewis gene (29, 30). In women who express an active form of the Secretor gene (approximately 70% of women), the most abundant HMOS is 2′-fucosyllactose (2’FL). Although 2’FL and 3FL are structurally alike, affinities for different receptors have been described (14) and although this has not been studied in depth, we hypothesize that their effects on the neonatal immune development may be distinguishable.

Currently it is unknown whether these HMOS may protect against allergic sensitization. Therefore, the aim of this study was to develop a human *in vitro* bronchial epithelial mucosal immune model to study the crosstalk between BEC, DCs and T cells after exposure to HDM, based on the models previously published for the intestinal mucosa (31–33). Furthermore, the potential immunomodulatory effects of the commonly expressed HMOS 2’FL and 3FL were investigated in this *in vitro* model. Additionally, an *in vivo* murine HDM-induced acute allergic asthma model was used to study the immunomodulatory effects of a dietary intervention with 2’FL and 3FL.

## 2 Materials and methods

### 2.1 *In vitro* culture and isolation of BEC, moDC, and T cells

#### 2.1.1 Calu-3 cell culture

Human lung adenocarcinoma Calu-3 cells (American Type Culture Collection, USA, passage 29–32) were used as a model for BEC. Cells were grown in minimal essential medium (MEM) (Gibco, USA) supplemented with 10% fetal bovine serum (FBS, Gibco), 1% penicillin, 1% streptomycin, 1% non-essential amino acids (Gibco), and 1% natrium pyruvate (Gibco) in a 75 cm^2^ culture flask (Greiner, Germany) until approximately 75% confluency was reached. After trypsinization, cells were seeded on inserts (3.0 μm pores) of a 24-wells transwell plate (Corning Incorporated, USA) in 200 μl medium. 500 μl medium was added to the basolateral compartment. The cells were incubated in 5% CO2 at 37°C. After 24 h (24 h) all medium was removed and only 300 μl basolateral medium was added to start air-liquid interface (ALI) culture. 200 μl was added to the apical compartment and 500 μl to the basolateral compartment for submerged culture. Medium was refreshed every 3–4 days. Cells were cultured for 2 weeks until 100% confluency, differentiation of the cells was assessed by measuring trans-epithelial electrical resistance (TEER) using the Locsense Artemis (Locsense, The Netherlands) or the Millicell ERS-2 Volt-ohm meter (Merck Millipore, USA).

#### 2.1.2 PBMC isolation

Buffy-coats were obtained from healthy donor blood from the Dutch Blood Bank. Human peripheral blood mononuclear cells (PBMC) were isolated by density gradient centrifugation (1,000 × *g*, 13 min) using Leucosep tubes (Greiner Bio-One, the Netherlands). The pellet was washed three times with PBS containing 2% FBS. To remove the remaining erythrocytes, lysis buffer was added (8.3 mg NH_4_HCl, 1 g KHC_3_O, 37.2 mg EDTA in 1 L demi water, sterile filtered) for 5 min. Cells were resuspended in 10 ml RPMI 1640 (Sigma-Aldrich, USA) supplemented with 10% FBS, 1% penicillin and 1% streptomycin. Obtained PBMCs were counted using the Z1 Coulter Particle Counter (Beckman Coulter, The Netherlands).

#### 2.1.3 Monocyte and naïve Th cell isolation

The PBMC suspension was centrifuged (300 × *g*, 10 min), the supernatant was discarded and the pellet resuspended in isolation buffer (2.5 g BSA, 2 mM EDTA in 500 ml PBS). Monocytes were isolated via negative selection using a QuadroMACS separator, LS columns and Monocyte Isolation kit II or naïve Th cells via a Naïve CD4+ T cell isolation kit (Miltenyi Biotec, Germany) according to manufacturer’s instruction. Collected flow through contained the enriched cell fraction and was centrifuged (300 x g, 10 min). Monocyte containing pellets were resuspended in RPMI 1640 supplemented with 10% FBS, 1% penicillin, and 1% streptomycin. Naïve Th cell containing pellets were resuspended in IMDM supplemented with 5% FBS, 1% penicillin, 1% streptomycin, 20 μg/ml apo-transferrine, and 50 μM *β*-mercaptoethanol. Cells were counted using the Z1 Coulter Particle Counter (Beckman Coulter). Isolated naïve Th cells were stored in 90% FBS and 10% DMSO in liquid nitrogen until further use.

#### 2.1.4 Monocyte derived dendritic cell culture

Monocytes were cultured in a 6 well plate (Greiner Bio-One) in RPMI 1640 supplemented with 10% FBS, 1% penicillin, and 1% streptomycin. Cytokines were added to a final concentration of 100 ng/ml IL4 and 60 ng/ml GM-CSF (ProSpec Bio, Israel) to induce differentiation into dendritic cells. Half of the medium and cytokines were refreshed every other day. Cells were cultured for 6 days before experimental use. After 6 days, moDCs were counted and diluted to a concentration of 1.5 × 10^6^ cells/ml for use in coculture experiments.

### 2.2 HDM dose response in BEC

After 14 days of culture in transwell, Calu-3 cells were apically exposed for 72 h to increasing doses of HDM extract (10–250 μg/ml, Greer Laboratories, USA). TEER was measured during this exposure period (t = 0 h, 1 h, 6 h, 12 h, 24 h, 72 h) and basolateral supernatants were collected after 24 h to measure cytokine secretion.

### 2.3 Airway epithelial mucosal immune coculture model

#### 2.3.1 Calu-3/moDC coculture

500 μl of moDC suspension (1.0×10^6^ cells/ml) was added to the wells of a 24-wells plate (Greiner Bio-One), if appropriate inserts containing confluent ALI-cultured Calu-3 cells were added. MoDCs were (basolateral) exposed to 0,01% or 0,05% 2’FL or 3FL (Carbosynth, UK) and incubated for 24 h at 37°C, 5% CO2. Next, epithelial cells or moDC were apically exposed to 10 μg/ml HDM (in 200 μl) and incubated for 24 h at 37°C, 5% CO2. Afterwards, basolateral supernatant was collected and stored at −20°C for cytokine measurement, moDCs were collected for analysis by flow cytometry or subsequent coculture with naïve Th cells.

#### 2.3.2 MoDC/Naïve Th cell coculture

5 × 10^4^ MoDCs were collected for subsequent coculture with naïve Th cells and were transferred to a 48-well culture plate (Greiner Bio-One) in 100 μl coculture medium (IMDM supplemented with 5% FBS, 1% penicillin, 1% streptomycin, 20 μg/ml apo-transferrin, and 50 μM β-mercaptoethanol). Isolated naïve Th cells were thawed and diluted to a concentration of 1.25 × 10^6^ cells/ml in coculture medium. 400 μl allogenic naïve Th cell suspension was added to the moDC suspension in the wells (1:10 DC:T cell ratio). Activation of naïve Th cells was aided by adding 5 ng/ml IL2 (ProSpec Bio) and 150 ng/ml anti-CD3 (BD Biosciences, USA) to the culture. The coculture was incubated for 5 days without medium refreshments. Cells were collected for flow cytometric analysis and supernatants were stored at −20°C for cytokine analysis.

### 2.4 *In vivo* house dust mite induced acute allergic mouse model

#### 2.4.1 Diet preparation

Experimental diets (produced by ssniff-Spezialdiäten GmbH, Germany) were based on an AING93 diet. This diet was adapted by addition of methionine and cysteine, and soy protein was used instead of casein. Furthermore, diets were supplemented with or without 0,5% or 1% 2’FL or 3FL (Jennewein GmbH, Germany). Supplementation of 2’FL and 3FL was isocaloric compensated with cellulose. Animals had ad libitum access to food and water, which were fully refreshed weekly.

#### 2.4.2 Animals

Six to seven week-old male BALB/cAnNCrl mice (Charles River, Germany) arrived at the animal facility of Utrecht University and were housed in individual ventilated cages with a 12 h/12 h light/dark cycle, controlled relative humidity (50–55%) and temperature (21 ± 2°C). Mice were randomly allocated to the experimental groups and housed with 3 animals per cage. Cage enrichment consisted of woodchipped bedding, wood curls (as nesting material) and a plastic shelter. This study was conducted in accordance with institutional guidelines for the care and use of laboratory animals of the Utrecht University, and all animal procedures were approved by the local Animal Welfare Body under an Ethical license provided by the national competent authority (Centrale Commissie Dierproeven, CCD), securing full compliance the European Directive 2010/63/EU for the use of animals for scientific purposes.

#### 2.4.3 Animal procedures

**Figure 1:**
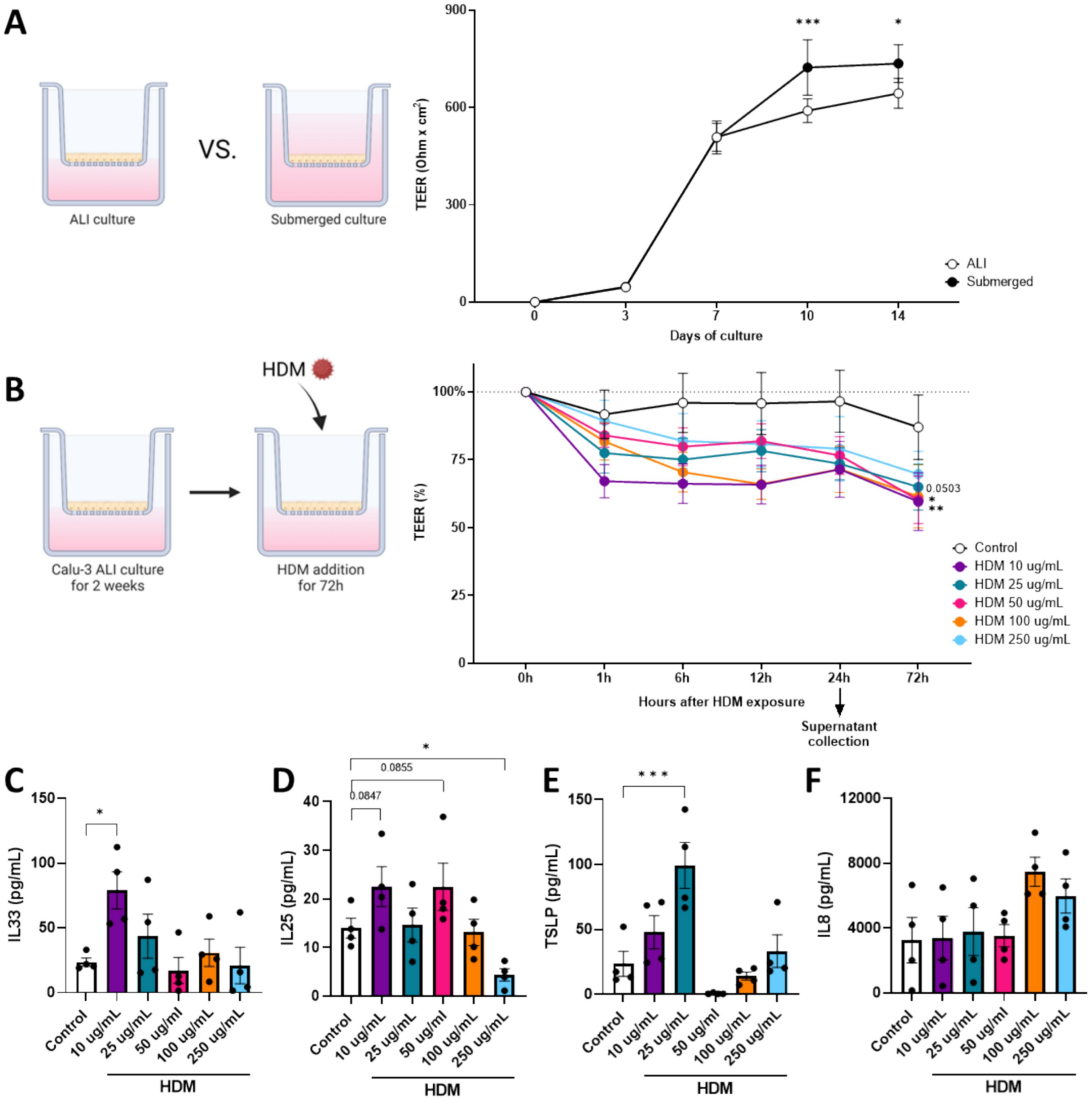
Dose response of HDM on Calu-3 cells to determine impact on barrier and cytokine secretion. In order to establish a physiological relevant *in vitro* model of bronchial epithelial cells, **(A)** Calu-3 cells were cultured in air-liquid interface (ALI) or submerged conditions. The development of transepithelial electrical resistance (TEER) of these cells was followed 14 days post seeding. After confirming that ALI-cultured Calu-3 cells establish appropriate TEER, **(B)** cells were exposed for 72 h to increasing dosages of HDM 14 days after seeding. After 24 h of HDM exposure, basolateral supernatant was collected to measure secretion of **(C)** IL33, **(D)** IL25, **(E)** TSLP and **(F)** IL8. Data is analyzed by One-Way or Two-Way ANOVA followed by a Dunnett’s multiple comparisons test, n = 4, mean ± SEM (* *p* < 0.05, ** *p* < 0.01, *** *p* < 0.001). Some parts of this figure were created with BioRender.com

A schematic overview of the experimental setup is shown in Figure 1A. After arrival mice immediately received the experimental diets. 14 days later, the mice were intranasally sensitized with 1 μg HDM (Greer Laboratories) in 40 μl PBS under isoflurane anesthesia. Mice were intranasally challenged on days 21–25 with 10 μg HDM in 40 μl PBS. 72 h after the final challenge, airway hyperresponsiveness was measured. Subsequently, mice were sacrificed by intraperitoneal overdose of pentobarbital (Nembutal™, Ceva Santé Animale, The Netherlands) and samples were collected for further analysis.

#### 2.4.4 Serum analysis

Mice were sacrificed and blood was collected via eyeball extraction in a Minicollect serum tube (Greiner Bio-One B.V., Netherlands). Collected blood was kept at room temperature for at least 30 min prior to centrifugation for 10 min at 14,000 rpm. Serum was collected and stored at −20°C for antibody measurement.

#### 2.4.5 Bronchoalveolar lavage

After sacrificing, lungs were lavaged four times with 1 ml saline solution (0.9% NaCl, 37°C). Bronchoalveolar lavage (BAL) fluid was centrifuged (400 × *g*, 5 min) and the BAL cell-containing pellets were pooled. Total cell count was determined using a Bürker-Türk chamber (magnification 100x). Cytospin preparations were made and stained with Diff-Quick (Merz & Dade A.G., Switzerland) for differential BAL cell count. Cell counts were scored with light microscopy.

#### 2.4.6 Preparation of lung homogenates

Collected lung tissue was homogenized using 1% Triton X100 (Sigma-Alrdrich) in PBS containing protease inhibitor (Complete Mini, Roche Diagnostics) with a Precellys Tissue Homogenizer and Precellys homogenizer tubes (Bertin, Rockville, MD, USA). Homogenates were centrifuged at for 10 min at 14000 rpm. Supernatant was collected and stored at −20°C for cytokine analysis.

#### 2.4.7 Preparation of lung single cell suspension

Lung tissue was collected after sacrifice and enzymatic digested using a buffer containing DNase I and Collagenase A (Roche Diagnostics, Switzerland). FBS was added to stop the digestion after 30 min. The lung tissue was passed through a 70 μm filter to obtain single cell suspensions. Cell suspensions were incubated for 4 min on ice in red blood cell lysis buffer (4.14 g NH_4_Cl, 0.5 g KHCO_3_, 18.6 mg NA_2_EDTA in 500 ml demi water, sterile filtered, pH 7.4). Lysis was stopped by adding FBS. Lung cells were washed with RPMI 1640 (Lonza, USA). After counting the cells with Z1 Coulter Particle Counter (Beckman Coulter Life Sciences), cells were stained for analysis by flow cytometry.

### 2.5 ELISAs

*In vitro* cytokine levels were determined in collected supernatants. IL4, IL8, IL10, IL13, IFNγ, TGFβ, TSLP (Invitrogen, USA), IL25 and IL33 (R&D systems, Minneapolis, MN, USA) were determined according to manufacturer’s protocol. Optical density was measured at 450 nm.

To measure HDM-specific IgE levels in serum from mice, high binding 96 well plates (Corning Costar) were coated with HDM (50 μg/ml) and incubated overnight at 4°C. After blocking with 1% BSA in PBS, plates were washed and diluted serum samples were added to incubate for 2 h. Plates were washed again and 1 μg/ml biotin anti-mouse IgE (BD Biosciences) was added for 1.5 h. Plates were incubated with streptavidin-HRP for 30 min, followed by addition of a substrate solution. Washing steps were performed in between. Reaction was stopped by addition of 2 M H2SO4 and absorbance was measured at 450 nm.

Concentrations of IL13, IFNγ and IL10 (Invitrogen) in lung homogenates were measured according to the manufacturer’s instructions. Levels of cytokines were calculated per mg of homogenized lung tissue.

### 2.6 Flow cytometry staining

Cells were stained with Fixable Viability Dye eFluor780 for 30 min. Aspecific binding was blocked using human FC block (BD Biosciences) in PBS for *in vitro* samples and antiCD16/CD32 blocking buffer for murine samples. Subsequently samples were stained for 30 min at 4°C using titrated amounts of antibodies. To allow intranuclear staining of transcription factors, cells were fixated and permeabilized using FoxP3/Transcription Factor staining buffer set (eBioscience, USA) following the manufacturer’s instructions. For intracellular staining, cells were fixated and permeabilized with Intracellular Fixation & Permeabilization Buffer Set (eBioscience) according to manufacturer’s protocol. FACS Canto II (BD Biosciences) was used to measure stained samples and obtained data was analysed using Flowlogic Software (Inivai Technologies, Australia).

Titrated amounts of the following antibodies were used to stain *in vitro* samples: CD11c-PerCP (3.9), HLA-DR-PE (LN3), CD80-FITC (2D10.4), CD86-PE/Cy7 (IT2.2) (All from eBioscience, USA), CD4-PerCP (OKTO4, eBioscience), CXCR3-Alexa Fluor 488 (1C6/CXCR3, BD Biosciences), CRTH2-APC (BM16, BD Biosciences), FoxP3-eFluor 660 (PCH101, Invitrogen), CD25-Alexa Fluor 488 (BC96, eBioscience), and IL13-PE (85BRD, eBioscience), and IFNγ-Amcyan (4S.B3, Biolegend). The gating strategy using representative samples is shown in Supplementary Figure 1.

Titrated amounts of the following antibodies were used to stain murine samples: CD4-BV510 (RM4-5, Biolegend), CD69-PE-Cy7 (H1.2F3, eBioscience), CXCR3-PE (CXCR3-173, eBioscience), T1ST2-FITC (DJ8, MD Bioproducts, USA), FoxP3-FITC (FJK-16 s, Invitrogen), CD127-PE/Vio770 (A7R 34, Miltenyi Biotech) and CD25-PerCP/Cy5.5 (PC61.5, Invitrogen).

### 2.7 Statistical analysis

Statistical analyses were performed using Graphpad Prism (Version 10.4.1) software. TEER data was analyzed by Two-Way ANOVA followed by Bonferroni multiple comparisons test. After exposure to increasing HDM concentrations, cytokine levels were analyzed by One-Way ANOVA followed by Dunnett’s multiple comparison test comparing all HDM concentration to the control condition. Control and HDM conditions (in presence or absence of BEC) were analyzed by paired t-test. When data fit the normal distribution and had an equal variability of differences between groups, effects of HMOS preincubations were analyzed by One-Way ANOVA followed by Dunnett’s multiple comparisons test comparing all conditions to the HDM-exposed condition. If these criteria were not met the following steps were taken in this order, data was transformed, a Geisser–Greenhouse correction was performed or a Friedman-test with Dunn’s multiple comparisons test was performed. Murine data was comparing Sham and HDM groups, and HDM group with the intervention groups. When data fit a normal distribution and had an equal variability of differences, Sham and HDM groups are analyzed by unpaired t-test, the intervention groups were compared to the HDM group by One-Way ANOVA followed by a Dunnett’s multiple comparisons test. When these criteria were not met, data was transformed, a Mann-Withney test or Kruskal-Wallis test with Dunn’s multiple comparisons test was performed. *p* < 0.05 is considered statistically significant, data is represented as mean ± SEM of 4–6 independent *in vitro* repeats using biologically different donors, or in case of *in vivo* studies using 6–12 animals per group.

## 3 Results

### 3.1 HDM exposure activates ALI cultured BEC and reduces barrier resistance

**Figure 2:**
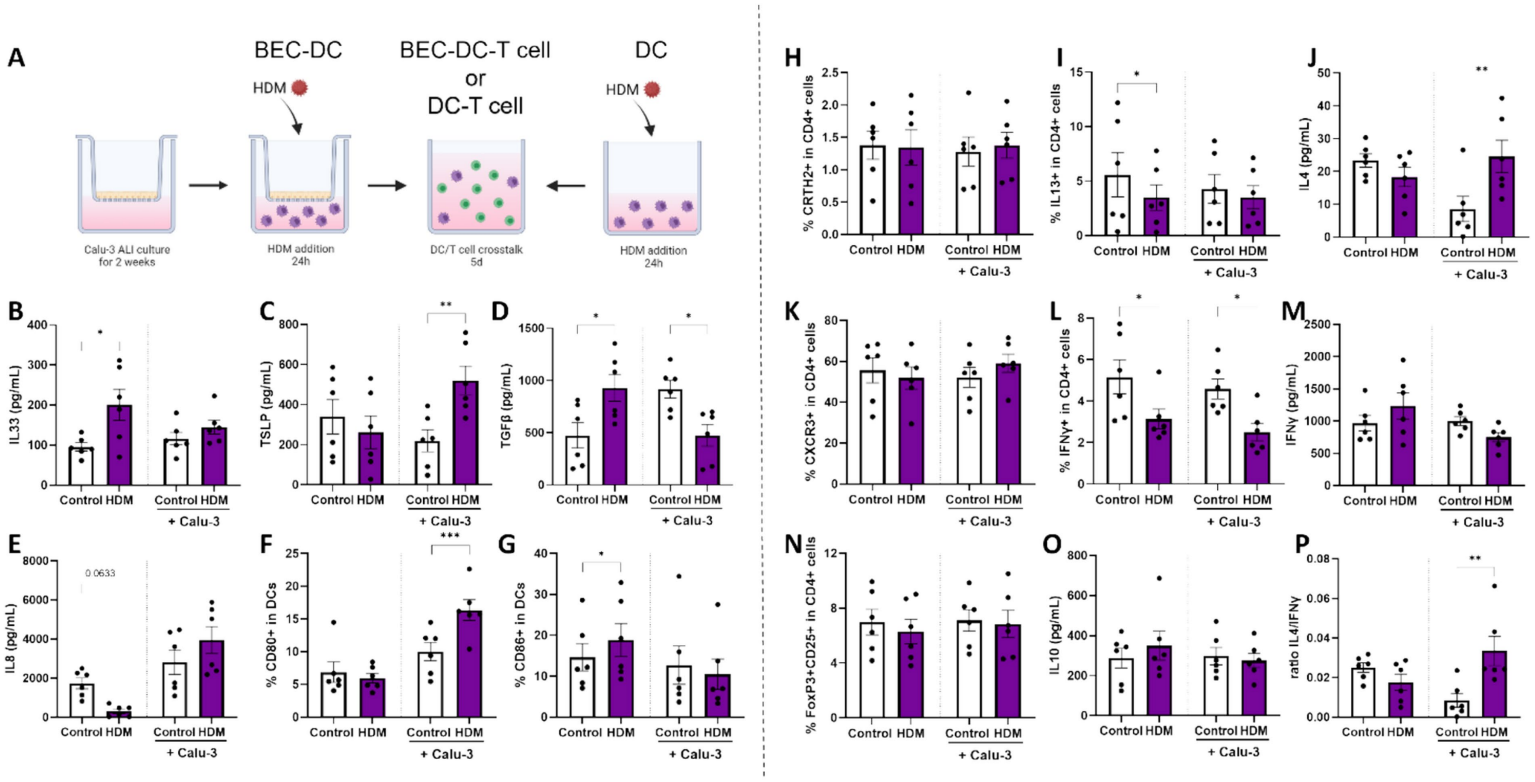
Mucosal immune response of BEC/DC/T cell upon HDM exposure. **(A)** A schematic overview of the coculture steps in this human in vitro bronchial mucosal immune model (adapted from (33)). Calu-3 bronchial epithelial cells (BEC) were cultured for 14 days in air-liquid interface (ALI) prior to coculture with monocyte-derived dendritic cells (moDCs). BEC-DCs and DCs alone were apically exposed to 10 μg/ml HDM for 24 h. After 24 h of exposure to HDM, primed DCs were collected for analysis and coculture with allogenic naïve Th cells for 5 days. Upon 24 h of HDM exposure, supernatants and moDCs were collected to measure secreted levels of **(B)** IL33, **(C)** TSLP, **(D)** TGFβ, **(E)** IL8 and determine the percentage of moDCs expressing the costimulatory markers **(F)** CD80 and **(G)** CD86. After subsequent coculture with of primed DCs with naïve Th cells, supernatants and cells were collected to measure the percentage of **(H)** CRTH2 and **(I)** IL13 expressing cells and secreted **(J)** IL4 as part of the T helper 2 cell response. In addition, the T helper 1 response was analyzed based on the percentage of **(K)** CXCR3 and (L) IFNγ expressing cells and secreted **(M)** IFNγ. The regulatory T cell response was determined by identification of the percentage of dual expressing **(N)** FoxP3 and CD25 cells and secretion of **(O)** IL10. Finally, **(P)** the ratio of type 2 IL4 and type 1 IFNγ secretion was calculated. Data is analyzed by paired *t*-tests, *n* = 6 biologically different donors (2 independent experiments each using 3 different donors), mean ± SEM (*p < 0.05, **p < 0.01, ***p < 0.001). Some parts of this figure were created with BioRender.com

The initial step to develop an *in vitro* airway epithelial mucosal immune model, consisted of a comparison between ALI and submerged cultured Calu-3 cells. During a culture period of 14 days after seeding, development of barrier resistance was measured by means of TEER. Although a significant difference was found after 10 and 14 days of culture, Calu-3 cells developed proper barrier resistance in the ALI culture (Figure 2A). Therefore all experiments using Calu-3 cells were performed in ALI culture. Next, a dose–response (10-250 μg/ml) HDM was added for 72 h. 10 μg/ml and 100 μg/ml HDM significantly decreased barrier resistance as measured already 1 h after exposure (Figure 2B), and a decreasing trend (*p* = 0.0503) was observed using 25 μg/ml HDM. Secreted cytokines were measured in basolateral supernatants collected 24 h after the start of HDM exposure. IL33 secretion was enhanced using 10 μg/ml HDM exposure (Figure 2C). Both 10 μg/ml (*p* = 0.0847) and 50 μg/ml (*p* = 0.0855) HDM exposure tended to increase IL25 levels, while 250 μg/ml HDM significantly decreased the release of IL25 (Figure 2D). TSLP secretion was significantly increased using 25 μg/ml HDM exposure (Figure 2E), whereas none of the HDM concentrations significantly enhanced IL8 secretion (Figure 2F). Based on these results, a dose of 10 μg/ml HDM was chosen to be used for further *in vitro* experiments.

The TEER upon HDM exposure was automatically measured by the Locsense Artemis. In subsequent experiments this decrease was not observed, possibly due to manual measurements performed by a Millicell ERS-2 Volt-ohm meter.

### 3.2 Exposing BEC-DC to HDM induces a Th2 profile during coculture with naïve Th cells

**Figure 3:**
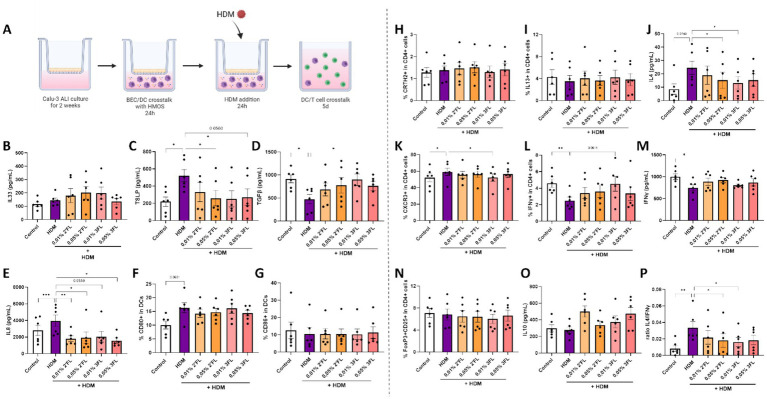
The immunomodulatory effects of HMOS were tested in this human in vitro bronchial mucosal immune model by preincubation of HMOS prior to HDM exposure. **(A)** Calu-3 BECs were cultured in ALI for 14 days before coculture with moDCs and basolateral preincubation with 0,01-0,05% 2’FL or 3FL for 24 h. Next, BEC/DC were apically exposed to 10 μg/ml HDM for 24 h. Followed by a coculture of primed moDCs with naïve Th cells for 5 days. After HMOS preincubation and HDM exposure, primed DCs were collected for analysis and coculture with allogenic naïve Th cells for 5 days (adapted from (33). Upon HMOS preincubation and HDM exposure, supernatants and moDCs were collected to measure secreted levels of **(B)** IL33, **(C)** TSLP, **(D)** TGFβ, **(E)** IL8 and determine the percentage of moDCs expressing the costimulatory markers **(F)** CD80 and **(G)** CD86. After subsequent coculture with of primed DCs with naïve Th cells, supernatants and cells were collected to measure the percentage of **(H)** CRTH2 and **(I)** IL13 expressing cells and secreted **(J)** IL4 as part of the T helper 2 cell response. In addition, the T helper 1 response was analyzed based on the percentage of **(K)** CXCR3 and **(L)** IFNγ expressing cells and secreted **(M)** IFNγ. The regulatory T cell response was determined by identification of the percentage of dual expressing **(N)** FoxP3 and CD25 cells and secretion of **(O)** IL10. Finally, **(P)** the ratio of type 2 IL4 and type 1 IFNγ secretion was calculated. When data fit a normal distribution and had an equal variability of differences, analysis was performed by One-Way ANOVA followed by a Dunnett’s multiple comparisons test. When these criteria were not met, data was transformed, a Geisser–Greenhouse correction was performed or a Friedman-test with Dunn’s multiple comparisons test was performed, *n* = 6 biologically different donors (2 independent experiments each using 3 different donors, mean ± SEM (*p < 0.05, **p < 0.01, ***p < 0.001). Controls of Figure 4 are the same as those depicted in Figure 3. Some parts of this figure were created with BioRender.com

BECs cocultured with moDCs (BEC-DC) or moDCs alone were exposed for 24 h to HDM (Figure 3A). moDC exposure to HDM in absence of BECs was found to increase IL33 and TGFβ secretion (Figures 3B,D) and the percentage of CD86 expressing DCs (Figure 3G), while the secretion of IL8 tended to be reduced (*p* = 0.0633, Figure 3E). By contrast, when BEC were exposed to HDM while cocultured with moDC, secretion of TSLP (Figure 3C) and the percentage of CD80 expressing DC (Figure 3F) was enhanced, while secretion of TGFβ (Figure 3D) was decreased in supernatants collected after 48 h BEC-DC coculture.

Subsequently, primed moDC were cocultured with allogenic naïve Th cells to study the functional character of the HDM-DC or HDM-BEC-DC. Although development of Th subsets was unaffected by any of the conditions (Figures 3H,K,N), HDM-DC reduced the percentage of IL13 or IFNγ containing T cells (Figures 3I,L). By contrast, HDM-BEC-DC enhanced the release of IL4 (Figure 3J) after coculture with naïve CD4+ T cells (HDM-BEC-DC/T), while decreasing the percentage of IFNγ containing Th cells (Figure 3L). Although the secretion of IFNγ, IL10 (Figures 3M,O), IL13, IL5 and IL17 (Supplementary Figures 3A-C) remained unaffected, the ratio of IL4 over IFNγ secretion was significantly increased for HDM-BEC-DC-T cells (Figure 3P), displaying the type 2 shifted immune character of HDM-BEC-DC-T cells. As the exposure to HDM via BEC resulted in a type 2 immune response, a relevant contribution of the BEC to the HDM-induced immune activation was hypothesized. Therefore, the latter model was chosen to explore the effects of specific HMOS.

### 3.3 Both 2’FL and 3FL reduce HDM mediated BEC-DC activation while consecutive BEC-DC/T cell responses are differentially modulated by these HMOS

**Figure 4:**
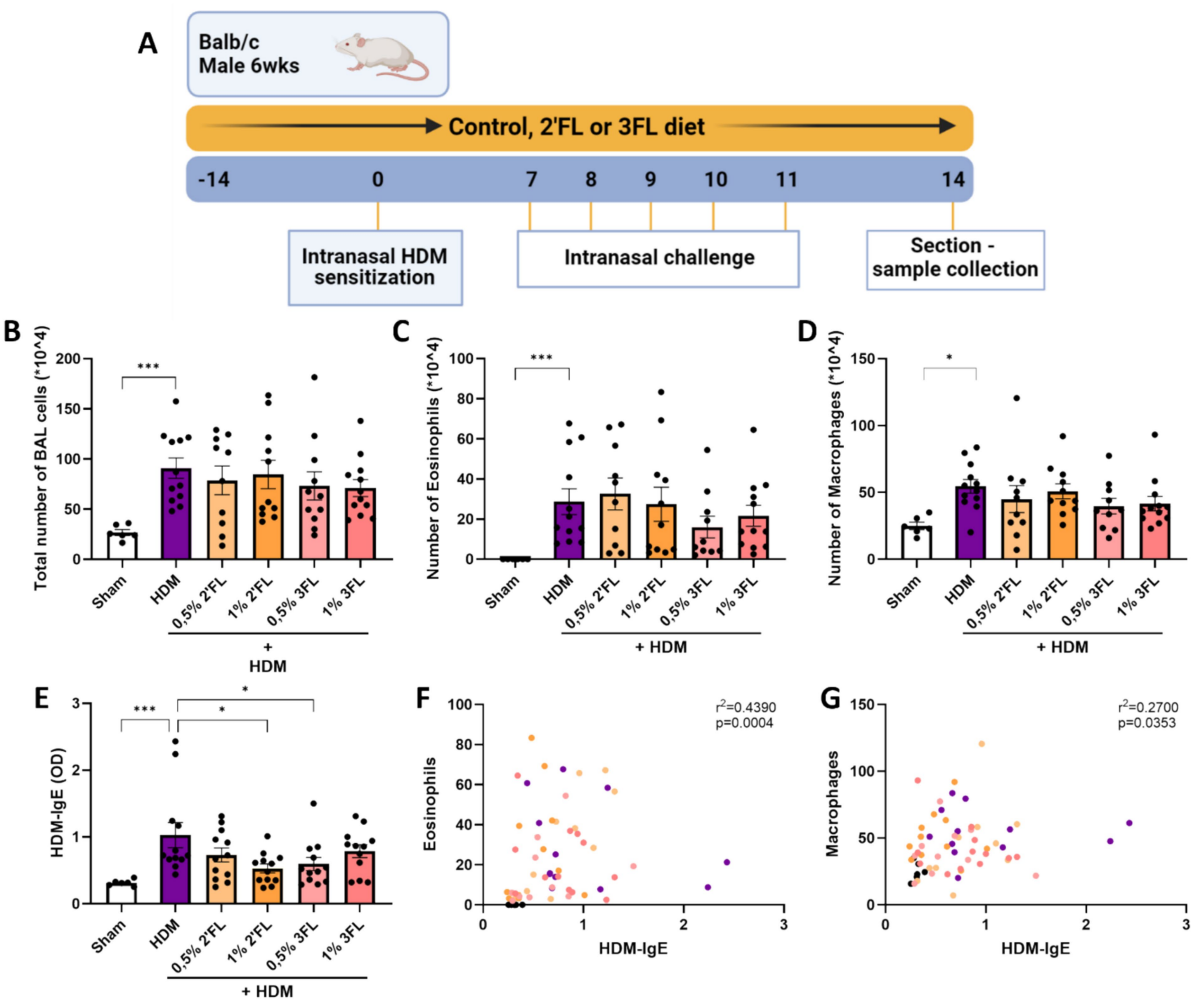
A preclinical murine model for house dust mite induced acute allergic asthma was used to investigate the immunomodulatory effects of dietary HMOS. **(A)** 6 week old male Balb/cAnNCrl mice were fed a control or HMOS supplemented diet 2 weeks prior to intranasal sensitization with HDM. One week after sensitization, mice were intranasally challenged for 5 consecutive days. 72 h after the final challenge, mice were sacrificed for sample collection. Lungs of sacrificed mice were lavaged to determine the influx of **(B)** the total number of cells, **(C)** eosinophils and **(D)** macrophages into the lungs. **(E)** HDM-specific IgE levels were determined in serum. The correlation between **(F)** eosinophil influx into the lungs and HDM-specific IgE in serum as well as **(G)** macrophage influx into the lungs and HDM-specific IgE in serum is displayed. When data fit a normal distribution and had an equal variability of differences, Sham and HDM groups are analyzed by unpaired t-test, the intervention groups were compared to the HDM group by One-Way ANOVA followed by a Dunnett’s multiple comparisons test. When these criteria were not met, data was transformed, a Mann-Withney test or Kruskal-Wallis test with Dunn’s multiple comparisons test was performed *n* = 6–12, mean ± SEM (*p < 0.05, **p < 0.01, ***p < 0.001). Correlations were studied using the Spearman’s correlation. Some parts of this figure were created with BioRender.com

The previously displayed Control and HDM conditions were compared to HDM exposed cells preincubated with 2’FL or 3FL. Although basolateral incubation of BEC-DC with 2’FL and 3FL showed only minor immunomodulatory effects on moDCs (Supplementary Figures 2A–F), no significant effects were observed when HMOS-primed moDCs were cocultured with naïve Th cells (Supplementary Figures 2G–Q). Next, BEC-DC were incubated basolateral with 2’FL and 3FL, while BEC were apically exposed to HDM. The BEC-DC were cocultured with naïve Th cells (Figure 4A). HDM induced TSLP and IL8 secretion (Figures 4C,E) was prevented when BEC-DC were incubated with 2’FL or 3FL. In addition, the HDM reduced TGFβ secretion was prevented by exposing the HDM-BEC-DC to 0,01% 3FL but not 2’FL (Figure 4D). IL33 levels or DC maturation were not significantly affected by either HDM exposure 2’FL or 3FL (Figures 4B,F,G). In these cultures TEER was measured, but HDM did not result in a TEER reduction (data not shown).

When combining BEC-DC with T cells, the 0,01% 3FL incubated HDM-BEC-DC were found to reduce the secretion of IL4 (Figure 4J) and IL13 (Supplementary Figure 3) from T cells. In addition, the percentage of Th1 cells, characterized by CXCR3 expression, (Figure 4K) was reduced but the IFNγ expression within the T cells was increased when comparing 0,01% 3FL preincubated HDM-BEC-DC-T cells to HDM-BEC-DC-T cells (Figure 4L). Although the secretion of IL5, IL17 (Supplementary Figures 3A-C) and IFNγ (Figure 4M) was not significantly affected by either 2’FL or 3FL preincubation, the ratio of IL4 over IFNγ was significantly reduced by 0,05% 2’FL as well as 3FL incubation of HDM-BEC-DC, indicating a shift in balance away from a type 2 immune response (Figure 4P). Furthermore, HDM exposure did not significantly affect the percentage of Treg cells or secretion of regulatory type IL10 (Figures 4N,O).

### 3.4 HDM sensitized and challenged mice receiving 1% 2’FL or 0,5% 3FL via diets have lower HDM-specific IgE levels in serum

To support the *in vitro* findings, a murine HDM-induced acute allergic asthma model was performed specifically to study the potential immunomodulatory effects of 2’FL and 3FL (Figure 1A). The influx of inflammatory cells into the lungs was increased in HDM-sensitized mice 72 h after the final challenge (Figure 1B). In particular, the number of eosinophils (Figure 1C) and macrophages (Figure 1D) was enhanced in HDM sensitized and challenged mice as compared to control. Mice receiving an HMOS supplemented diet had similar levels of cell influx into the lungs. No significant increase in airway hyperresponsiveness was observed in HDM sensitized and allergic mice (Supplementary Figure 4). As hallmark parameter of allergic sensitization, HDM-specific IgE levels were measured in serum. HDM sensitized and challenged mice had increased levels of HDM-specific IgE, which was reduced in mice who had received a 1% 2’FL or 0,5% 3FL diet (Figure 1E). Even though the influx of eosinophils and macrophages was not significantly altered in mice receiving an HMOS supplement diet, the number of both eosinophils and macrophages was positively correlated with the levels of HDM-specific IgE in serum (Figures 1F,G).

### 3.5 Lower levels of pulmonary IL13 and IFNγ in HDM sensitized and challenged mice receiving 1% 3FL containing diets

**Figure 5:**
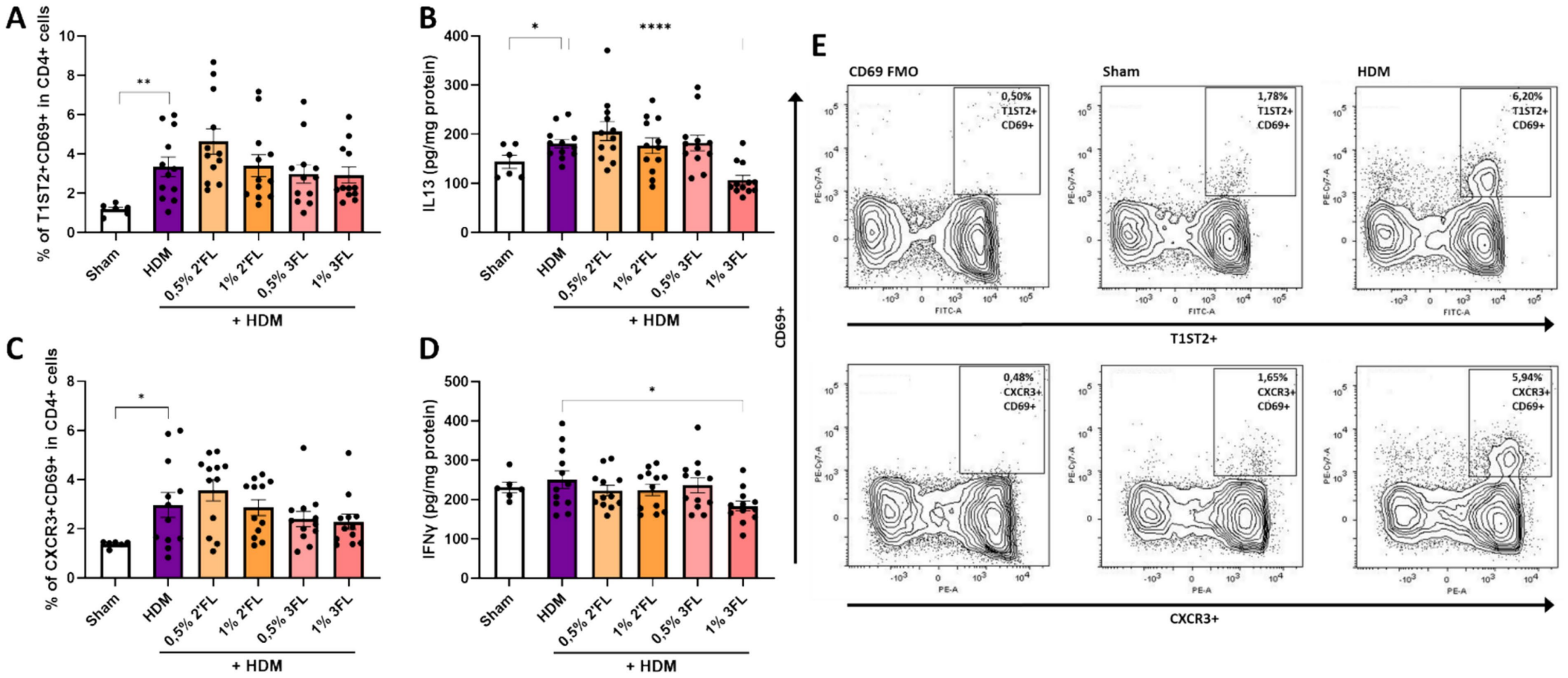
T cell responses in lung tissue. Lung tissue of sacrificed mice was collected to determine the population of **(A)** activated T helper 2 cells based on the expression of T1ST2 and CD69 and the concentration of **(B)** IL13 in lung tissue as well as the population of **(C)** activated T helper 1 cells based on the expression of CXCR3 and CD69 and the concentration of **(D)** IFNγ in lung tissue. **(E)** Representative flow cytometry samples from Sham and HDM mice. When data fit a normal distribution and had an equal variability of differences, Sham and HDM groups are analyzed by unpaired t-test, the intervention groups were compared to the HDM group by One-Way ANOVA followed by a Dunnett’s multiple comparisons test. When these criteria were not met, data was transformed, a Mann-Withney test or Kruskal-Wallis test with Dunn’s multiple comparisons test was performed, *n* = 6–12, mean ± SEM (*p < 0.05, **p < 0.01, ***p < 0.001, ****p < 0.0001).

Lung tissue from sacrificed mice were analyzed for the ratio of present Th cell subsets and cytokine levels. HDM sensitized and challenged mice had a higher percentage of activated Th2 and Th1 cells present (based on expression of T1ST2 and CXCR3, respectively, as well as CD69, Figures 5A,C,E), however these percentages were not altered in the mice receiving either 2’FL or 3FL in their diets. The percentage of regulatory T cells (Treg) remained the same in all HDM sensitized and challenged mice (Supplementary Figure 4B). However, levels of IL13, IFNγ (Figures 5B,D) and IL10 (Supplementary Figure 4C) were lowered in homogenized lung tissue from mice that had received a 1% 3FL enriched diet as compared to mice receiving a control diet.

## 4 Discussion

HMOS are the third most abundant solid component of human milk, they may act as prebiotics and are believed to have immunomodulatory properties. This may help to prevent allergic diseases such as type 2 asthma. Therefore, the immunomodulatory properties of two commonly expressed fucosylated HMOS, 2’FL and 3FL, were investigated in an *in vitro* bronchial epithelial HDM induced type 2 mucosal immune model and a murine model for HDM-induced acute allergic asthma.

To the best of our knowledge, this is the first study on the development and use of an *in vitro* bronchial epithelial mucosal immune model using HDM as the allergenic trigger. This model is based on a previously model for the intestine (31–33). It is known that epithelial cells play a key role in initiating the allergic sensitization process by influencing the maturation and activation of DCs (34). Therefore, Calu-3 cells were used as model for BEC. Although traditional transwell culture methods are fully submerged (35, 36), ciliated characteristics of the bronchial epithelium are diminished, resulting in less relevant physiological conditions. Furthermore, Calu-3 cells are known to produce mucus which is a relevant feature in airway barrier function (37). ALI culture is suggested to reflect a more representative condition as the cells are exposed to medium via the basolateral compartment and air via the apical compartment resembling the physiological airway conditions (38, 39). Even though the TEER development of ALI cultured BEC was significantly lower compared to submerged cultured BEC, the ALI cultured BEC still acquired a high degree of barrier resistance and therefore this culture method was selected for the succeeding experiments.

It is known that HDM can disrupt bronchial epithelium integrity via proteolytic breakdown of tight junction proteins (38, 40–42). In the current study, we observed that the TEER was significantly decreased already at a relatively low apical HDM exposure (10 μg/ml) within 1 h after exposure. Exposure to the lowest HDM dose was also sufficient to enhance release of alarmins, known to drive a subsequent type 2 response via DCs. Next, the immunological crosstalk between BEC and DC was studied during HDM exposure and compared to HDM exposure of DC alone, in addition to the subsequent functional T cell response after coculture with HDM-DC or HDM-BEC-DC was investigated. In the absence of BEC, HDM exposed DC showed increased IL33 and TGFβ secretion together with enhanced CD86 expression, while IL8 secretion was reduced. IL33 is a type 2 instructing epithelial derived alarmin, which can be produced by DCs when stimulated via Dectin-1. From murine experiments, it is known that HDM binding to Dectin-1 is crucial for DC migration, Th2 development and allergic airway inflammation (43). Although IL33 is mainly known for its proinflammatory, type 2 driving properties, this cytokine can induce the development of Treg cells as well (44). Furthermore, increased expression of CD86 by DCs has been linked to promote the development of an allergic type 2 response (45, 46). Yet in the presence of regulatory TGFβ this enhanced CD86 expression was linked to a tolerogenic phenotype in which IL8 secretion was decreased as well (47). This may imply that HDM exposure to DC may instruct a more regulatory phenotype. However, subsequent coculture of HDM-DCs with naïve Th cells had no significant effect on Treg cell development nor regulatory cytokine secretion (IL10). Yet the developed T cells did show lower intracellular expression of both IL13 as well as IFNγ, indicating some immunosuppressive effects which may relate to the phenotypic changes of HDM-DC. Previously, it was demonstrated that exposing DCs from healthy donors to HDM did not result in subsequent activation of naïve Th cells, while DCs from allergic patients promoted Th2 cell polarization upon HDM exposure (48). These differences in DC responses to HDM between allergic and healthy individuals may explain the observations in this study and highlights the relevance to further investigate the role of DCs in allergic diseases.

However, when BEC were exposed to HDM while cocultured with DC, secretion of TSLP and expression of CD80 on DC was enhanced, while TGFβ concentrations decreased. Although CD80 has been linked to a type 1 and Treg cell instruction (45), we hypothesize that due to the increase in TSLP and decrease in TGFβ the functional outcome of DC development was altered, leading to DCs that directed the consecutive T cell response towards a type 2 prone cytokine pattern. HDM-BEC-DC instructed T cells to increase the release of IL4, and the ratio of IL4 over IFNγ cytokine secretion, while a lower percentage of Th cells contained intracellular IFNγ when compared to medium controls. Epithelial-derived TSLP promotes DCs to induce Th2 differentiation resulting in IL4 secretion (49–51). In addition, TGFβ stimulates the development of tolerogenic responses while inhibiting the production of proinflammatory cytokines (52). Hence, the increased secretion of TSLP and reduced TGFβ secretion seemed sufficient to develop a type 2 directed DC response, affecting the subsequent coculture with naïve Th cells, as generally observed in asthma allergic patients. These results emphasize the relevance of further studying the crosstalk between BEC and DCs in shaping the mucosal immune response to aeroallergens.

After the observation that HDM only induced a type 2 driven immune response in presence of BEC, the immunomodulatory effect of 2’FL and 3FL were investigated in the presence of BEC, thus using the BEC-DC-T model. Prior to HDM exposure, cocultured BEC and moDC were preincubated with a low dose of these HMOS in the basolateral compartment, mimicking the physiological concentrations that can become systemically available via the bloodstream after ingestion of HMOS in infants (24–26). Both 2’FL as well as 3FL prevented the HDM-induced release of TSLP and IL8. IL8 secretion from DC can be triggered in the presence of TSLP (53), justifying the similar secretion patterns observed in these cytokines. However, in this setting IL8 may also have been derived from BEC. The general decrease in HDM induced TSLP and IL8 secretion from BEC-DC after HMOS preincubation was associated with a reduction in HDM increased IL4 over IFNγ secretion by the T cells which were cocultured with the BEC-DC. Hence both HMOS not only suppressed HDM induced type 2 activation in BEC-DC cultures, it also prevented the instruction of type 2 prone cytokine secretion by T cells exposed to DC from these cultures. These data suggest that the HMOS are able to prevent HDM-induced TSLP secretion and hereby may be able to modify DC function to steer the subsequent T cell response away from a type 2 reaction, which is provoked by HDM when exposed to BEC (54, 55). Although here BEC-DC cocultures were incubated 24 h with HMOS, another physiological relevant approach for future studies would be to prime the moDCs with these low doses of HMOS already during their differentiation from monocyte into DC.

HMOS naturally occur at a 1–2.5% concentration in human milk. 2’FL and 3FL are generally the dominant HMOS present in human milk. *In vitro* we studied the dose of 0.01 and 0.05%, since it is estimated that 1–2% of the HMOS in milk could become available systemically (56). Interestingly, only 0,01% 3FL preincubation restored the HDM-induced decrease of TGFβ secretion during BEC-DC coculture. During subsequent coculture of BEC-DC with T cells, 0,01% 3FL preincubation also significantly reduced HDM-BEC-DC instructed IL4 secretion by T cells, while preventing the decrease in percentage of IFNγ expressing cells as observed in the HDM-BEC-DC/T condition. TGFβ is known for its regulatory functions, such as promoting the development of Treg while suppressing Th1 and Th2 responses (57). Here, 0,01% 3FL preincubation mediated enhanced secretion of TGFβ in BEC-DCs resulting in restoration of the HDM-induced type 1 and type 2 response in T cells, while Treg differentiation remained unaffected. Previously, we demonstrated that both 2’FL and 3FL preincubation of intestinal epithelial cells prior to ovalbumin exposure directed moDC to induce enhanced release of IL10 by T cells in an *in vitro* intestinal mucosal immune model for ovalbumin-induced type 2 inflammation (58). Hence, both 2’FL and 3FL may be able to directly affect epithelial cells and/or immune cells and in this way suppress type 2 activation, while permitting regulatory cytokine secretion. In general not much information is available on systemic HMOS concentrations, future dose-finding studies are warranted also including lower concentrations (24). In addition, future studies should aim to combine different HMOS based on their relative presence in human milk (59) to study the optimal composition for immune support.

To further study the potential immunomodulatory effects of 2’FL and 3FL, a murine model for HDM-induced acute allergic asthma was used. Mice received a control, 2’FL or 3FL containing diet 2 weeks prior to and during HDM sensitization and challenge. Previously, this murine model was used to study other prebiotic dietary interventions (60, 61). It was demonstrated that diets containing 1% w/w galacto-oligosaccharides prevent the influx of inflammatory cells into the lungs in HDM sensitized and challenged mice (60, 62), which was also demonstrated for a high-pectin fiber diet (63). In the current study, HDM sensitized and challenged mice did not show signs of increased airway hyperresponsiveness (Supplementary Figure 4A). The influx of inflammatory cells was significantly increased in HDM-allergic mice, yet dietary intervention with 2’FL or 3FL did not decrease the influx of cells into the lungs significantly. However, the mice receiving 1% 2’FL or 0,5% 3FL containing diets had lower HDM-specific IgE levels in serum, indicative of reduced allergic sensitization towards HDM. Although this did not significantly protect against airway eosinophilia, a declining pattern was observed in the mice fed 3FL. This may associate with the reduced levels of HDM-IgE in this group, since HDM-IgE and the number of eosinophils were positively correlated. The same may apply for the macrophages, however here the correlation with HDM-IgE was less clear. Changes in dietary fiber or prebiotics intake indeed can affect allergy related humoral responses, as was previously demonstrated in HDM sensitized mice receiving a low-fiber diet that had higher levels of total IgE in serum, compared to HDM sensitized mice fed a high-fiber diet (63).

The 2’FL or 3FL containing diets did not affect the tendency of HDM to induce activation of Th1 and Th2 cells. Nonetheless, mice receiving the 1% 3FL containing diet had lower levels of both IL13 and IFNγ present in lung tissue, suggesting an additional anti-inflammatory capacity of 3FL compared to 2’FL which was also observed in the *in vitro* studies. Reduced cytokine levels were also observed in lung homogenates of HDM sensitized and challenged mice being fed GOS/lcFOS or scFOS/lcFOS plus *Bifidobacterium breve* M16V supplemented diets compared to control diet (64). Of note: the HMOS used in the *in vitro* studies were enzymatically derived from lactose. However, due to limitations in production capacity, the HMOS used in the *in vivo* studies were produced in genetically modified *E. coli*. Previously, we demonstrated that the source of HMOS (bacterial or enzymatic produced) affects the immunomodulatory effects in different types of *in vitro* immunoassays (65). Future studies should take into account the possible translational difference between lactose derived versus *E.coli* derived HMOS on immunomodulatory efficacy. Even though single 2’FL or 3FL did not protect against HDM induced eosinophilic airway inflammation, the *in vivo* results do indicate 2’FL or 3FL to protect against HDM allergic sensitization as was predicted by the *in vitro* model.

Our current knowledge of the mechanisms underlying HMOS mediated immunomodulation are poorly understood. However, several potential routes have been described, including both direct and indirect immunomodulatory effect, via which HMOS may prevent allergic sensitization (65). Future studies should focus on exploring the underlying mechanisms via which HMOS exert their immunomodulatory effect. This will contribute to understanding structure–function relationships of HMOS and their role in immunomodulation as relevant in early life immune development.

## 5 Conclusion

In this study, we established an *in vitro* coculture model for HDM induced type 2 BEC-DC activation and subsequent development of a type 2 Th cell response, which resembles the *in vivo* situation. BEC necessary in vitro to induce type 2 immunity after HDM exposure via modulation of DC functionality. Preincubation of BEC-DC with 2’FL and 3FL largely prevented HDM-induced BEC-DC activation and suppressed downstream type 2 over type 1 T cell activation. Although both 2’FL and 3FL reduced HDM-specific IgE in a murine model for HDM allergic asthma, these mice were not protected against airway inflammation. Future studies should consider improvement of *in vitro* models from a 3R perspective to further investigate the potential allergy preventive effects of early life influencing factors.

## Data Availability

The raw data supporting the conclusions of this article will be made available by the authors, without undue reservation.
