# Supplementary material for: HMOS 2’FL and 3FL prevent house dust mite induced proinflammatory cytokine release in vitro and decrease specific IgE production in a murine allergic asthma model
Source: Front Nutr. 2025 Feb 19;12:1491430. doi: 10.3389/fnut.2025.1491430 (PMC11879794; doi:10.3389/fnut.2025.1491430)
Supplement: Supplementary file 1 [file Image_1.pdf]

Supplementary Material

1 Supplementary Figures and Tables

1.1 Supplementary Figures

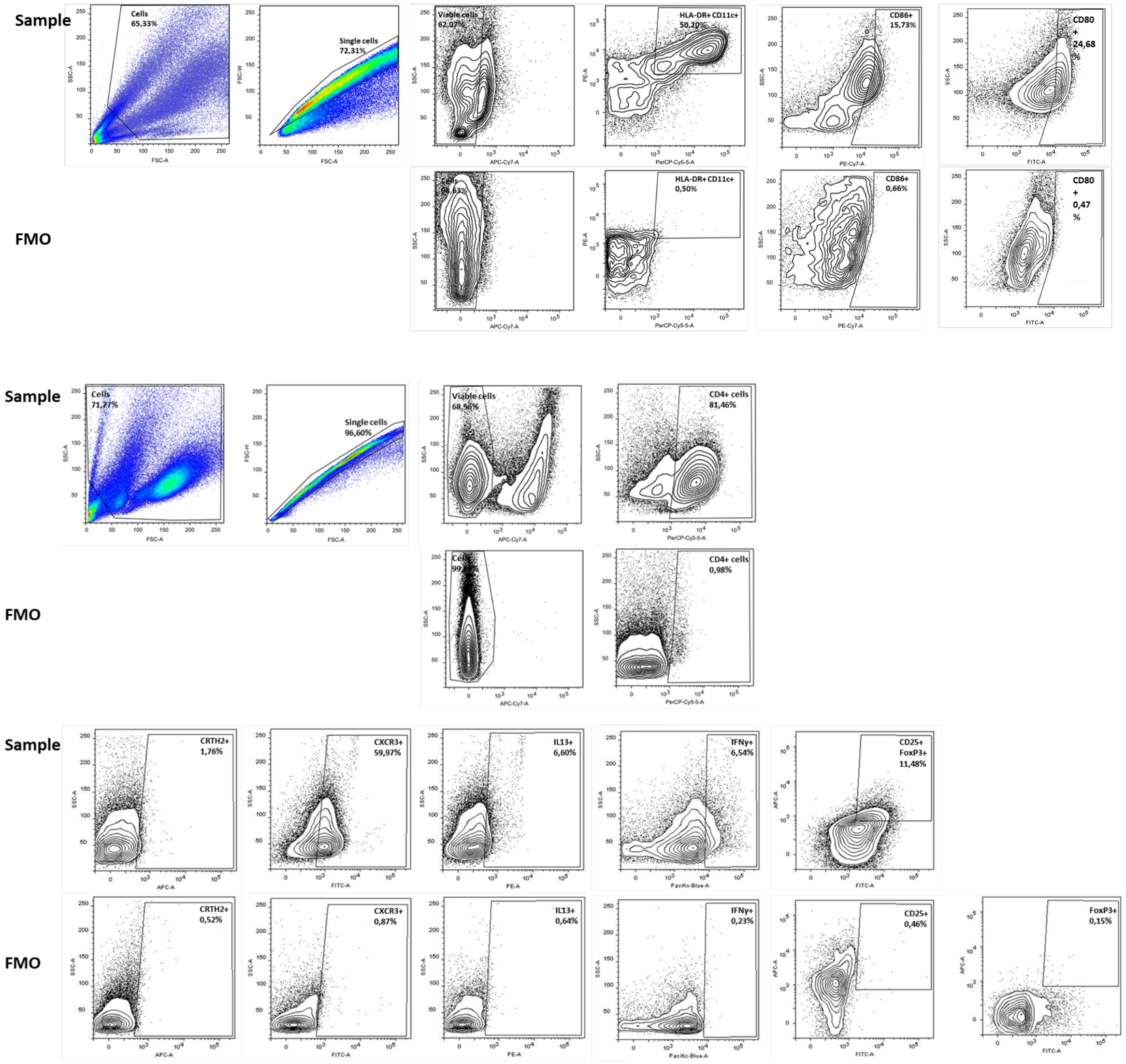

Supplementary Figure 1. FACS gating strategy of DCs and T cells from the human in vitro bronchial mucosal immune model.

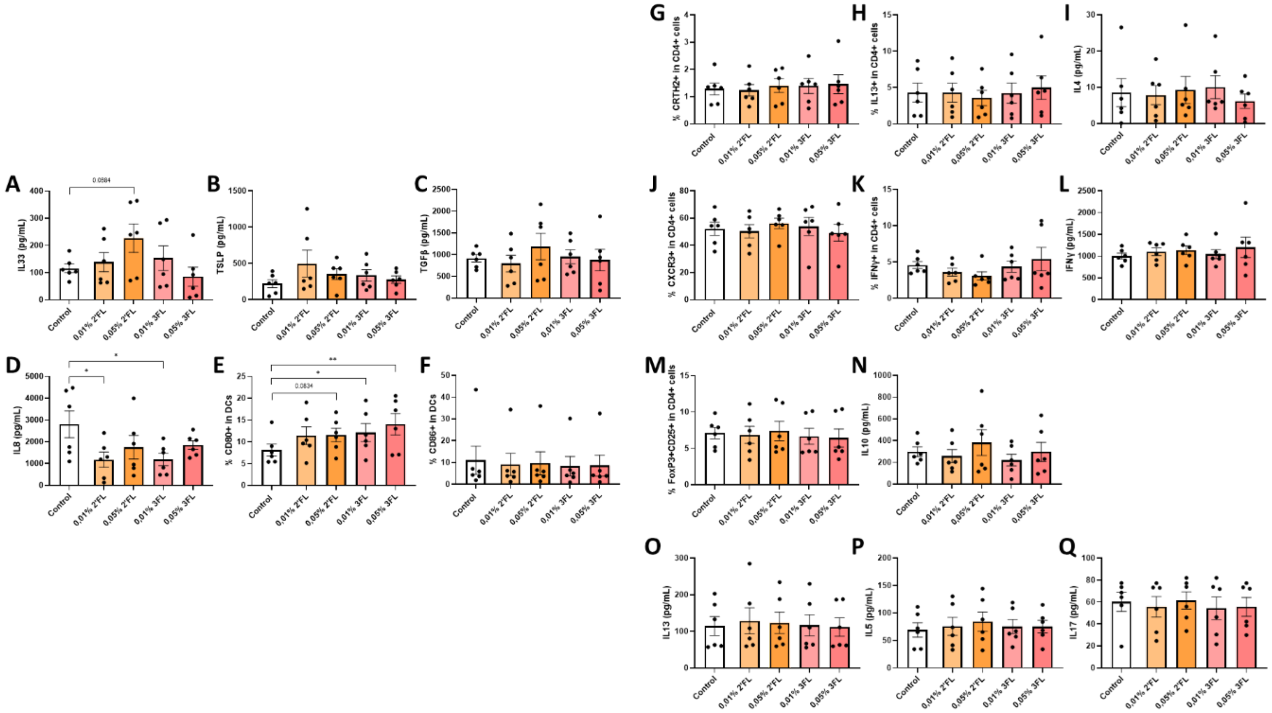

Supplemental Figure 2. The immunomodulatory effects of HMOS were tested *in vitro* in the bronchial mucosal immune model. A) Calu-3 BECs were cultured in ALI for 14 days before coculture with moDCs and basolateral preincubation with 0,01-0,05% 2'FL or 3FL for 48h. After HMOS incubation, primed DCs were collected for analysis and coculture with allogenic naïve T cells for 5 days. Upon HMOS incubation, supernatants and moDCs were collected to measure secreted levels of A) IL33, B) TSLP, C) TGFβ, D) IL8 and determine the percentage of moDCs expressing the costimulatory markers E) CD80 and F) CD86. After subsequent coculture with of primed DCs with naïve T cells, supernatants and cells were collected to measure the percentage of G) CRTH2 and H) IL13 expressing cells and secreted I) IL4 as part of the T helper 2 cell response. In addition, the T helper 1 response was analyzed based on the percentage of J) CXCR3 and K) IFNγ expressing cells and secreted L) IFNγ. The regulatory T cell frequency was determined by identification of the percentage of dual expressing M) FoxP3 and CD25 cells and secretion of N) IL10. Finally, secretion of O) IL13, P) IL5 and Q) IL17 were determined. Data is analyzed by One-Way ANOVA followed by a Dunnett's multiple comparisons test, n=6 biologically different donors (2 independent experiments each using 3 different donors, mean ± SEM (* $p<0.05$, ** $p< 0.01$, *** $p<0.001$).

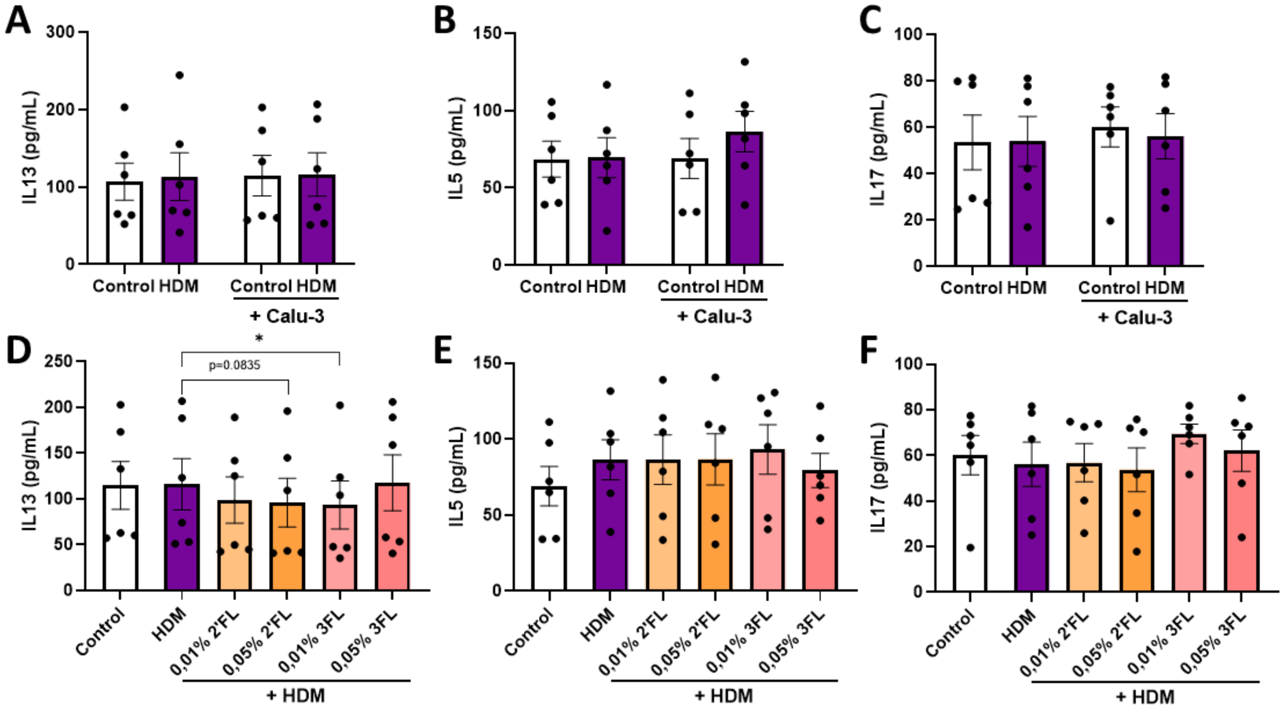

Supplemental Figure 3. Cytokine levels from supernatants collected after DC-T cell coculture upon HDM and HMOS exposure. Concentrations of A,D) IL13, B,E) IL5 and C,F) IL17 were determined by ELISA. Data is analyzed by One-Way ANOVA followed by a Dunnett's multiple comparisons test, n=6 biologically different donors (2 independent experiments each using 3 different donors, mean ± SEM (* p<0.05, ** p< 0.01, *** p<0.001). Control of Supplemental Figure 3 D,E,F are the same as those depicted in Figure 3 A,B,C.

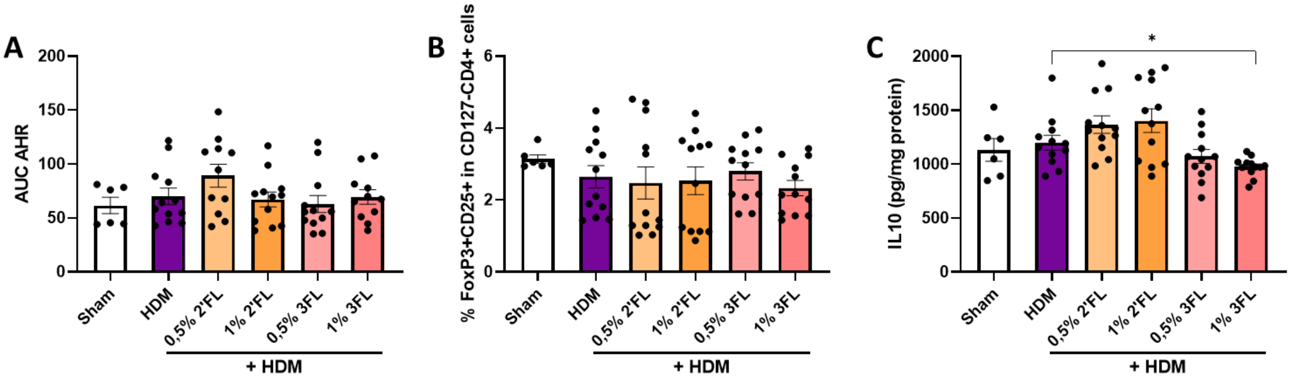

Supplemental Figure 4. 72h after the final challenge, mice were terminally anesthetized to measure airway hyperresponsiveness upon methacholine exposure. A) Area under the curve of the airway resistance after exposure to increasing dosages of methacholine (0,39-50mg/mL) compared to baseline was calculated. The population of regulatory T cells was determined in lung tissue based on B) expression of FoxP3 and CD25. C) Presence of IL10 in lung tissue was determined by ELISA. Data is

analyzed by One-Way ANOVA followed by a Dunnett's multiple comparisons test, n=6-12, mean ± SEM (* $p<0.05$, ** $p< 0.01$, *** $p<0.001$).
